# Single-cell RNA-seq combined with bulk RNA-seq explores shared gene signatures between thyroid and breast cancers

**DOI:** 10.3389/fgene.2025.1609189

**Published:** 2025-11-17

**Authors:** Zhiping Feng, Liang He, Xin Yang, Anhao Wu, Jingnan Wang, Yuanhua Song, Yongchun Zhou

**Affiliations:** 1 Department of Nuclear Medicine, The Third Affiliated Hospital of Kunming Medical University, Kunming, Yunnan, China; 2 Department of Medical Laboratory, The Third Affiliated Hospital of Kunming Medical University, Kunming, Yunnan, China; 3 Department of Blood Transfusion, The First People’s Hospital of Yunnan Province, Kunming, Yunnan, China; 4 Department of Breast Surgery, The Third Affiliated Hospital of Kunming Medical University, Kunming, Yunnan, China; 5 Department of Oncology, Kunming Children’s Hospital of Kunming Medical University, Kunming, Yunnan, China; 6 Center for Molecular Diagnostics, The Third Affiliated Hospital of Kunming Medical University, Kunming, Yunnan, China

**Keywords:** breast cancer, thyroid cancer, shared hub gene, therapeutic targets, PILRa, MKI67, UBE2C

## Abstract

**Objective:**

This study aims to identify key genes that are common to both breast cancer and thyroid cancer, as well as to determine shared therapeutic targets relevant to both conditions.

**Methods:**

We utilized transcriptome data from both breast and thyroid cancers, along with single-cell data, and applied cell deconvolution techniques to evaluate the extent of monocyte infiltration. Tumor-related gene modules were identified through weighted gene co-expression network analysis (WGCNA), followed by enrichment analysis to uncover significant signals shared within these gene modules. A machine learning approach was then employed to pinpoint hub genes. Additionally, RT-qPCR was performed to validate the expression levels of these hub genes in tumor and adjacent non-tumor tissues from patients with both cancer types.

**Results:**

Our analyses revealed that the transcriptional networks of breast cancer and thyroid cancer display significant similarities. WGCNA identified two consensus modules that are strongly associated with both cancers and monocyte infiltration. Enrichment analysis highlighted glycosaminoglycan synthesis pathways as critical signals that are common to both cancers. A total of seven hub genes were identified using the machine-learning approach. Results from RT-qPCR and immunohistochemistry in clinical samples showed that the expression levels of PILRA, Mki67, and UBE2C were markedly different between cancerous and adjacent tissues.

**Conclusion:**

PILRA, MKI67, and UBE2C, as potential diagnostic and prognostic biomarkers, are anticipated to serve as promising therapeutic targets for the clinical management of both breast cancer and thyroid cancer.

## 1 Introduction

Breast cancer (BC) and thyroid cancer (TC) are among the most prevalent malignant tumors in females (Roman et al., 2017). According to World Health Organization (WHO) statistics from 2023, approximately 2.3 million new BC cases are diagnosed globally each year (Arnold et al., 2022). The incidence of BC is consistently higher in women than in men. Due to its high malignancy and strong metastatic potential, BC remains a major challenge in terms of treatment and prognosis (Bray et al., 2018). In contrast, TC has exhibited one of the fastest-growing incidence rates among all cancers over the past two decades (Vigneri et al., 2015), affecting approximately 66 individuals per 1 million population worldwide (Siegel et al., 2016). Both age and gender are important prognostic factors for TC, with women having a threefold higher incidence compared to men (Mazzaferri, 1991). Notably, clinical evidence suggests that patients with TC are at increased risk of developing secondary BC, and TC is reported to be the most common second primary malignancy among BC survivors (Muller and Barrett-Lee, 2020). These observations imply the existence of shared etiological factors, suggesting that BC and TC may act as mutual risk factors for one another.

Over the past decade, a growing body of research has provided compelling evidence of a bidirectional pathogenic relationship between breast cancer and thyroid cancer. Several studies have indicated that the prevalence of thyroid nodules is higher in breast cancer patients compared to the general population (Ikeda et al., 2016). Additionally, research has shown that breast cancer patients are at an elevated risk of developing thyroid disease both prior to and following the diagnosis of breast cancer, in comparison to individuals with other malignancies. Furthermore, individuals with hypothyroidism have a higher likelihood of developing breast cancer than those with normal thyroid function (Ortega-Olvera et al., 2018). Notably, as early as 2013, Van et al. evaluated data from the American Cancer Society and demonstrated that female thyroid cancer patients had a 0.67-fold increased risk of subsequent breast cancer, while the incidence of thyroid cancer in female breast cancer patients was found to be twofold higher. More strikingly, male thyroid cancer patients were found to have a 29-fold increased risk of developing breast cancer, and male breast cancer patients exhibited a 19-fold increased risk of developing thyroid cancer (Van Fossen et al., 2013). Research suggests that thyroid and estrogen signaling pathways may serve as pathogenic factors for both cancers (Nielsen et al., 2016). Both estrogen receptors (ERα) and thyroid-stimulating hormone receptors (TSHR) belong to the G protein-coupled receptor (GPCR) family, which can activate similar signaling cascades (e.g., via cAMP/PKA, MAPK) to mediate biological effects. Additionally, estrogen itself has been shown to influence thyroid function. This shared hormonal dependency implies that both tissue types may exhibit molecular similarities in their sensitivity to changes in the hormonal microenvironment (Bakos et al., 2021). Despite the wealth of studies investigating the independent risk factors and treatment strategies for breast and thyroid cancers, there is a notable paucity of research addressing the common risk factors and potential therapeutic targets shared by both conditions.

This study integrates transcriptomic and single-cell sequencing data from breast and thyroid cancers to perform differential expression analyses, with the aim of identifying genes and cell types that exhibit concordant alterations in both malignancies. WGCNA was subsequently applied to identify gene modules associated with these cell types. Machine learning algorithms were then employed to pinpoint the hub gene, which was further examined through regulatory network analysis to identify its associated microRNAs. Collectively, this multi-level approach seeks to uncover shared molecular targets and candidate therapeutics for the treatment of breast and thyroid cancers.

## 2 Materials and methods

### 2.1 Flow chart

**FIGURE 1:**
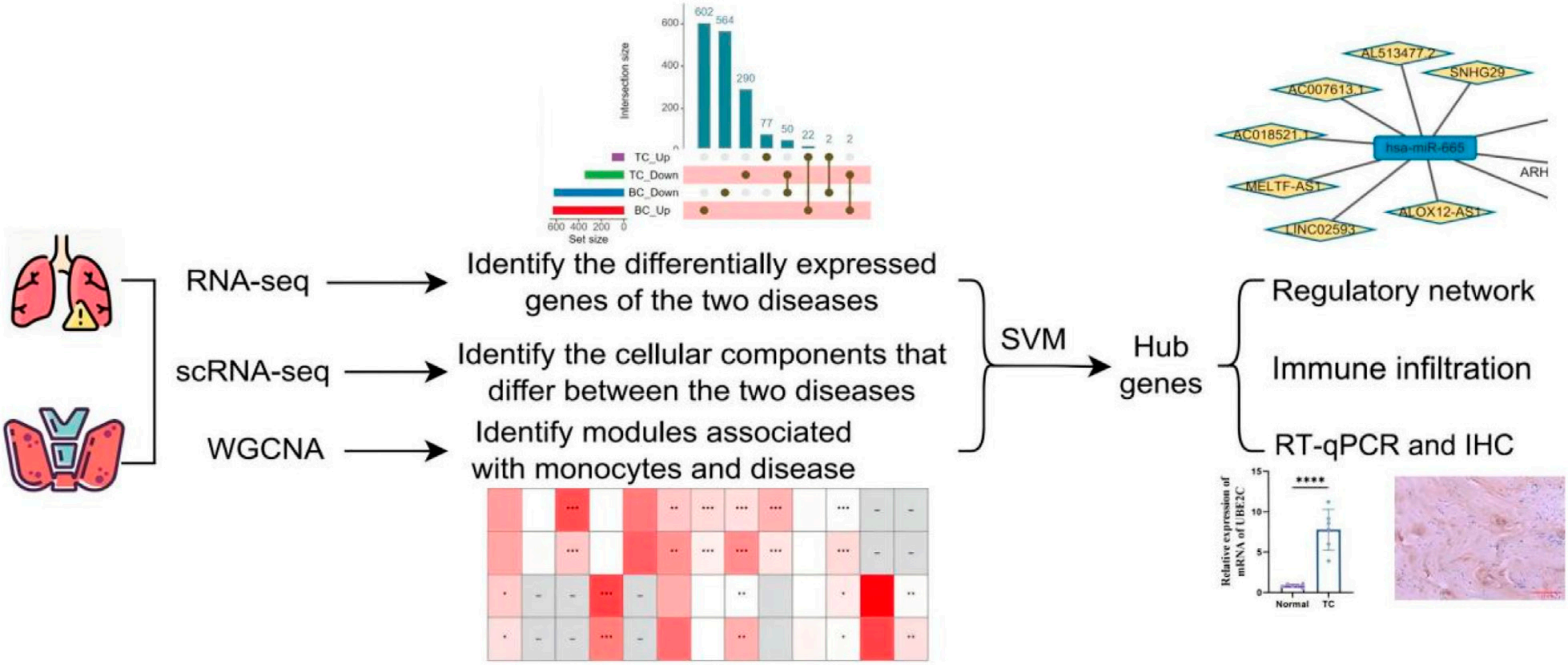
Flowchart of the analysis used in this study.

This study integrates transcriptomic and single-cell sequencing data from BC and TC, and employs a multi-faceted, multi-method analytical approach to identify shared molecular targets for the treatment of BC and TC (Figure 1).

### 2.2 Data sources

Transcriptomic data (RNA-seq) for BC and TC were obtained from the Gene Expression Omnibus (GEO) database (https://www.ncbi.nlm.nih.gov/geo/). Specifically, GSE124646 (which includes 90 breast cancer samples and 10 normal samples) and GSE126698 (comprising 22 thyroid cancer samples and 6 normal samples) were used for the primary data analysis. For validation of hub genes, GSE109169 (containing 25 breast cancer samples and 25 normal samples) and GSE140109 (which includes 6 thyroid cancer samples and 4 normal samples) were utilized.

Single-cell transcriptomic data (scRNA-seq) for BC and TC were also sourced from the GEO database. The datasets included GSE161529, which contains 6 breast cancer samples and 13 normal samples, and GSE191288, which comprises 6 thyroid cancer samples and 1 normal sample.

### 2.3 Differential gene analysis and functional enrichment analysis

Differentially expressed genes (DEGs) between cancer and normal samples were identified using the limma package in R, with the thresholds set to adjusted P < 0.05 and |log_2_FC| > 0.585. Subsequently, the ClusterProfiler package was employed to perform Kyoto Encyclopedia of Genes and Genomes (KEGG) pathway and Gene Ontology (GO) enrichment analyses on the identified DEGs, followed by visual representation of the results. The enrichment analysis parameters were set to pvalueCutoff = 0.05 and qvalueCutoff = 0.5.

### 2.4 scRNA-seq data analysis

Quality control of the single-cell transcriptomic datasets was performed using the Seurat R package (v4.1.2). Low-quality cells and low-expression genes were excluded based on the following criteria: the number of detected features per cell ranged from 200 to 5,000, the number of transcripts per cell ranged from 1,000 to 20,000, and the proportion of mitochondrial gene expression per cell was less than 20%. Data normalization was carried out using the NormalizeData function, and highly variable genes were identified using the FindVariableFeatures function with nfeatures = 2000.

To correct batch effects, the Harmony R package (version 0.1.1) was applied. Subsequently, linear transformation was performed using the ScaleData function. Classification was carried out through Principal Component Analysis (PCA) and Partial Least Squares Discriminant Analysis (PLS-DA) models (Chen et al., 2021). The optimal number of principal components (PCs) was assessed using the ElbowPlot function, and 50 PCs were selected for further analysis. Cell clustering was performed using the FindNeighbors and FindClusters functions (dims = 1:30, resolution = 2).

Cell clusters were annotated based on canonical marker genes reported in the literature. DEGs among cell types were identified using the FindAllMarkers function in Seurat, with the parameters min. pct = 0.1 and logfc. threshold = 0.25, retaining only genes with p < 0.05.

Additionally, to estimate the cellular composition from bulk RNA-seq data, the CIBERSORT function in the IOBR R package was applied for deconvolution analysis, with the permutation count (perm) set to 100.

### 2.5 WGCNA analysis

Weighted Gene Co-expression Network Analysis (WGCNA) was performed using the WGCNA R package (v1.72-1) to identify gene modules correlated with BC and TC phenotypes. Differentially expressed genes from the GSE124646 (BC) and GSE126698 (TC) datasets were used to construct gene expression matrices for network analysis. To determine the appropriate soft-thresholding power (β) required for scale-free topology, the pickSoftThreshold function was applied to both datasets, with optimal β values ranging from 10 to 12. Subsequently, an adjacency matrix was computed using the formula (aij = |Sij|β). The adjacency matrix was then transformed into a topological overlap matrix (TOM), and a dissimilarity matrix (1−TOM) was calculated. Hierarchical clustering based on this dissimilarity matrix was performed to identify distinct gene modules. Modules with strong correlations to clinical phenotypes were selected as candidate modules for downstream analyses, including biomarker discovery and functional annotation.

### 2.6 Shared hub gene screening and verification

The “randomForest” R software package was used to use the random forest (RF) machine learning algorithm to screen central genes that are highly related to thyroid cancer and breast cancer. The classification accuracy of different numbers of RF feature genes was determined, and the feature genes with the highest classification accuracy were retained to determine the final Hub genes. In order to test the diagnostic efficacy of Hub genes, the receiver operating characteristic (ROC) curve and the corresponding area under the ROC curve (AUC) of each Hub gene were calculated based on the normalized expression level of each Hub gene.

### 2.7 Immune cell infiltration analyses and its correlation with hub genes

The CIBERSORT algorithm was applied to the GSE126698 (TC) and GSE124646 (BC) datasets to estimate the relative proportions of 22 immune cell types within each sample. Differences in immune cell composition between cancer and normal tissues were assessed using the Wilcoxon rank-sum test, with P values calculated for statistical significance. To further evaluate tumor microenvironment (TME) characteristics at the sample level, the ESTIMATE R package was employed to calculate the immune infiltration score (ImmuneScore), stromal cell content (StromalScore), composite microenvironment score (ESTIMATEScore), and tumor purity (TumorPurity). Spearman’s rank correlation analysis was conducted to assess the association between hub gene expression and immune cell infiltration levels. A P value <0.05 was considered statistically significant.

### 2.8 TF regulatory network and miRNA network analysis of hub genes

Tumor-related microRNAs (miRNAs) were retrieved from the Human miRNA Disease Database (HMDD) (http://www.cuilab.cn/hmdd). Hub gene-associated mRNA–miRNA interaction pairs were obtained from the miRWalk database (http://mirwalk.umm.uni-heidelberg.de/), and intersected with the tumor-related miRNAs to identify relevant mRNA–miRNA regulatory relationships. Only pairs with a target score >80 were retained for further analysis. To identify potential long non-coding RNAs (lncRNAs) interacting with the tumor-associated miRNAs, predictions were performed using the ENCORI database, and corresponding lncRNA–miRNA interaction pairs were collected.

For transcriptional regulatory analysis, the RcisTarget R package was used to predict transcription factors (TFs) targeting the hub genes (Aibar and González-Blas, 2017). Motif enrichment analysis was conducted to identify significant TF-binding motifs, which were then used to construct the TF regulatory network.

### 2.9 RT-qPCR

Fresh tumor tissues and corresponding adjacent normal tissues were collected from six patients diagnosed with both TC and BC, ensuring the integrity and freshness of all specimens. Each sample was rinsed with physiological saline and homogenized into a tissue suspension. Total RNA was extracted using TRIzol reagent according to the manufacturer’s protocol, followed by DNase treatment to eliminate genomic DNA contamination. Reverse transcription was performed using a commercial reverse transcription kit to synthesize cDNA. For qRT-PCR, 2 μL of cDNA was placed into an EP tube and amplified using a SYBR Green pre-mix. GAPDH was used as the internal reference gene. The threshold cycle (Ct) values were determined, and relative mRNA expression levels were calculated using the 2^−ΔΔCT^ method.

### 2.10 Immunohistochemistry

Tissues from six patients with concurrent TC and BC, including tumours and adjacent normal tissues, were fixed with 4% paraformaldehyde, then embedded in paraffin and sectioned. The sections were stained according to the manufacturer’s instructions to observe the expression levels of relevant proteins in the sections.

### 2.11 Statistical analysis

All calculations and statistical analyses in this study were performed using R (https://www.r-project.org/, version 4.1.2). To determine the statistical significance of differences between two groups of normally distributed data, an independent Student’s t-test was used, while the Mann-Whitney U test (i.e., Wilcoxon rank-sum test) was employed to assess differences between non-normally distributed variables. All p-values were calculated based on two samples, and p-values less than 0.05 were considered statistically significant. Additionally, Spearman correlation analysis was used in this study to obtain the correlation coefficients between variables. P-values were calculated on both sides of the equation, and values less than 0.05 were considered statistically significant.

Immunohistochemistry and RT-qPCR data were analysed using SPSS 26.00 statistical software. All data are presented as mean ± standard deviation. A non-parametric t-test was used to compare the two groups of data. A p-value <0.05 was considered statistically significant.

## 3 Results

### 3.1 Identification of common differentially expressed genes (Co-DEGs) in breast and thyroid cancer

**FIGURE 2:**
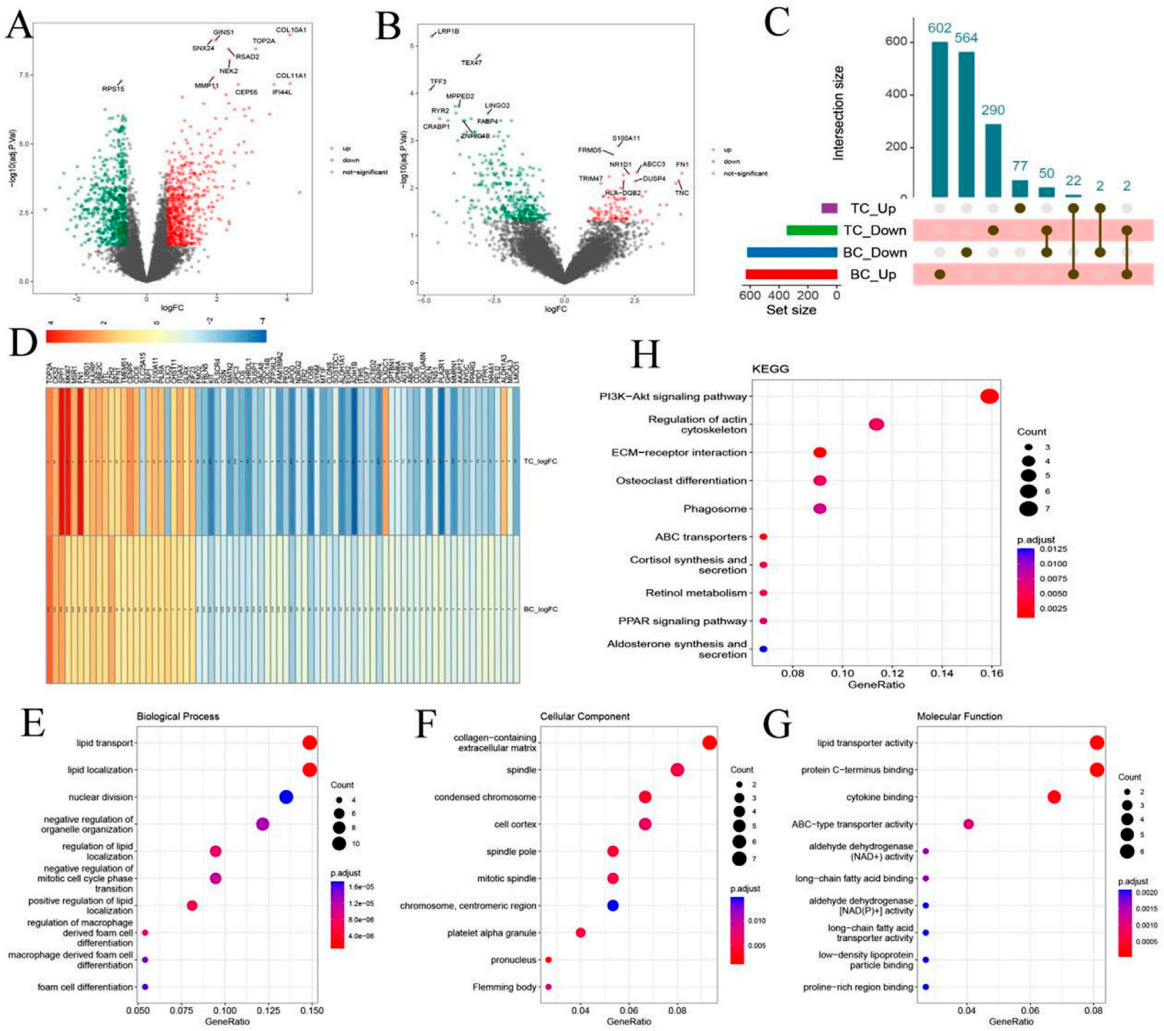
Identification of Common Differentially Expressed Genes (Co-DEGs) in Breast and Thyroid Cancer. **(A)** Volcano plot shows DEGs between healthy samples and breast cancer samples; **(B)** Volcano plot shows DEGs between healthy samples and thyroid cancer; **(C)** Overlapping DEGs between breast cancer and thyroid cancer; **(D)** Co-DEG in Differential change folds under different diseases; **(E–G)** GO enrichment analysis of Co-DEG; **(H)** KEGG results of Co-DEG.

Differential expression analysis of the BC dataset identified a total of 1,242 dysregulated genes, including 626 upregulated and 616 downregulated genes (Figure 2A). In the TC dataset, 101 genes were upregulated and 342 were downregulated (Figure 2B). Cross-comparison of the two datasets revealed 76 overlapping genes (Co-DEGs) shared between BC and TC (Figures 2C,D). Among them, 50 genes were consistently downregulated, 22 consistently upregulated, and 4 displayed divergent expression trends between the two cancers. GO and KEGG pathway enrichment analyses indicated that the Co-DEGs were predominantly involved in ECM-receptor interaction, ABC transporter regulation, and cortisol synthesis and secretion (Figures 2E–H).

### 3.2 Identification of shared cellular components in breast and thyroid cancer

**FIGURE 3:**
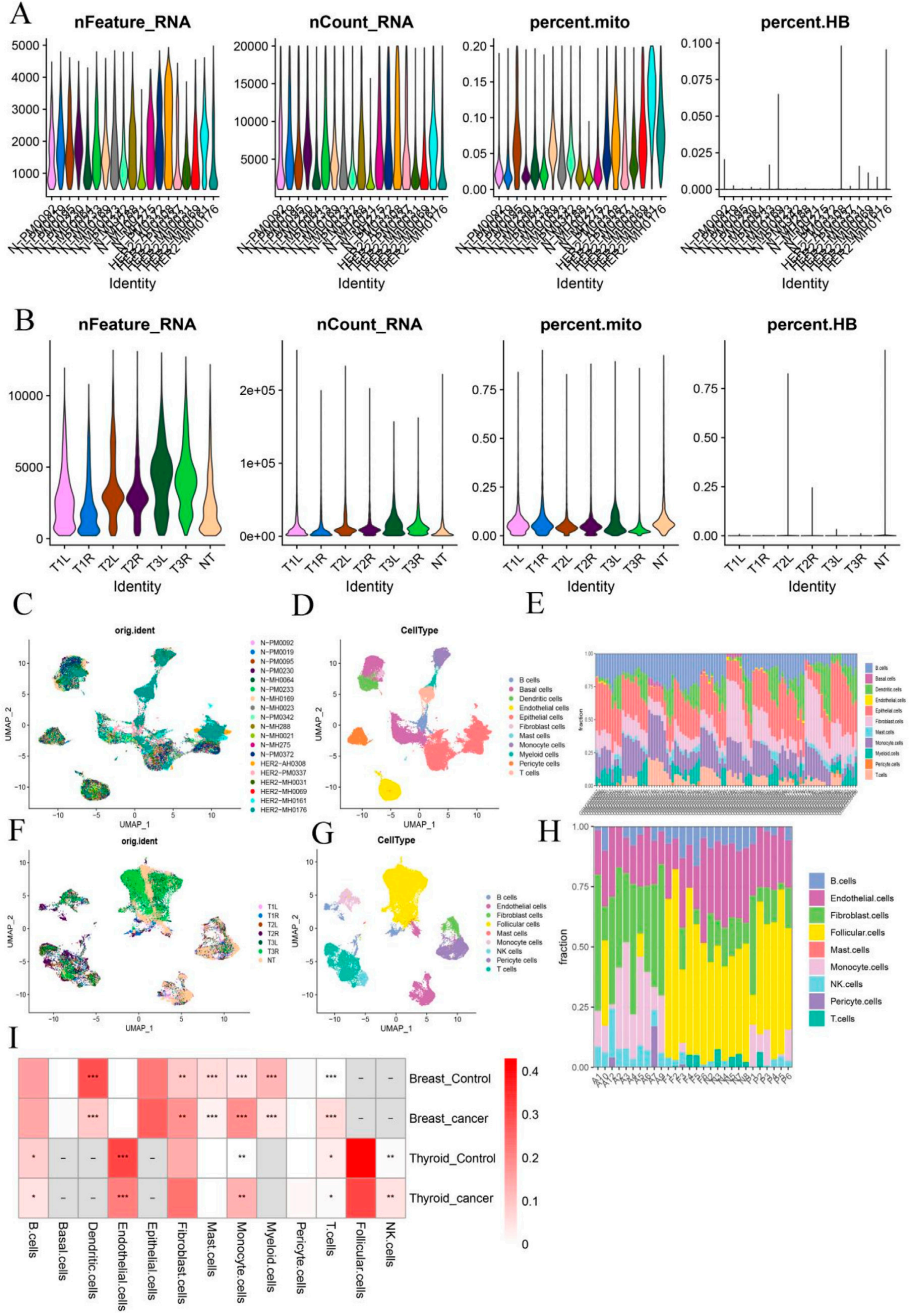
Identification of Shared Cellular Components in Breast and Thyroid Cancer **(A,B)** Quality control chart of BC and TC; **(C,F)** UMAP chart of batch samples from two sets of data sets; **(D,G)** UMAP annotation of different cell types from two sets of data sets; **(E,H)** Histogram of cell type proportion in samples after reverse convolution of transcriptome data; **(I)** Heat map of the proportion of each cell type in cancer samples versus healthy samples. *:p < 0.05; **:p < 0.05; ***:p < 0.001.

To explore shared cellular components between BC and TC, we integrated scRNA-seq data and deconvolution analysis. scRNA-seq datasets GSE161529 (BC) and GSE191288 (TC) were analyzed separately. Following quality control (Figure 3A) and batch effect correction (Figure 3C), 75,069 cells from the BC dataset were classified into 11 distinct cell types (Figure 3D), and deconvolution analysis confirmed significant variation in these cell types across samples (Figure 3E). In the TC dataset, 32,735 cells were classified into 9 cell types (Figures 3B,F,G), and similarly, transcriptome deconvolution revealed significant heterogeneity across TC samples (Figure 3H). Notably, both BC and TC patients exhibited increased monocyte infiltration compared to healthy controls (Figure 3I). This observation led to a focus on monocyte infiltration in subsequent analyses.

### 3.3 Identification of shared gene modules via WGCNA

**FIGURE 4:**
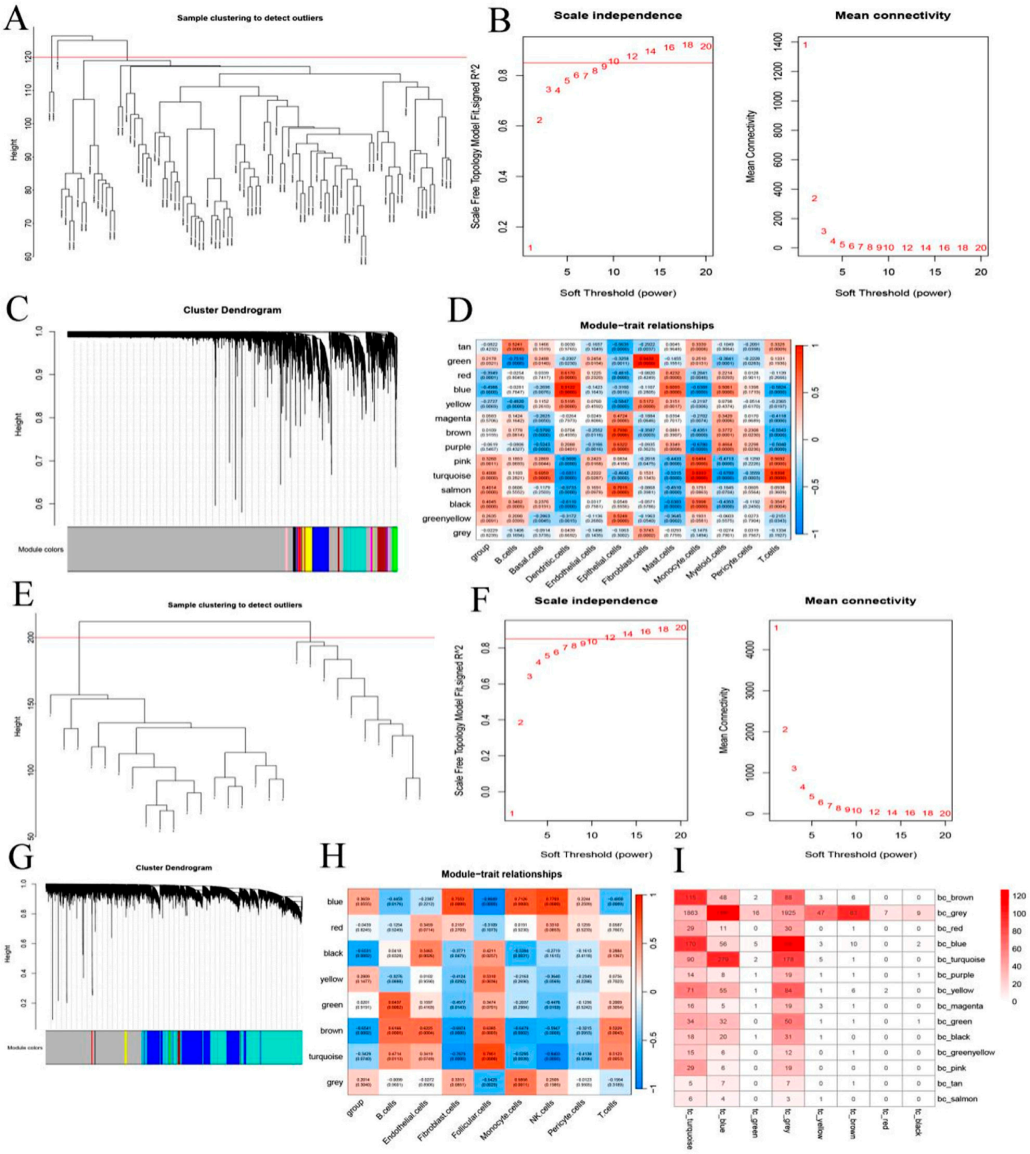
Identification of Shared Gene Modules via WGCNA. **(A,E)** Indicates the sample clustering diagram; **(B,F)** Indicates the scale-free fitting index and average connectivity of 1–20 soft threshold power (β); **(C,G)** Clustered tree diagram; **(D,H)** Heat map representing the correlation between characteristic factors and phenotypes of each module; **(I)** Number of overlapping genes in the breast cancer module and thyroid cancer module.

To identify gene expression modules shared by BC and TC, WGCNA was performed. For the BC dataset, a soft-threshold power of 10 was selected to ensure scale-free topology (Figures 4A,B), resulting in the identification of 14 co-expression modules, each represented by a unique color (Figure 4C). Module–trait relationships were assessed to identify modules significantly associated with BC phenotypes (Figure 4D). Similarly, WGCNA was applied to the TC dataset using a soft-threshold power of 12 (Figures 4E,F), which yielded 8 distinct modules (Figure 4G). The correlation between each module and TC phenotypes was also analyzed (Figure 4H). To assess cross-cancer module conservation, we evaluated the overlap between BC-specific and TC-specific modules. The analysis revealed substantial overlap, indicating shared transcriptional networks between BC and TC (Figure 4I).

Moreover, consistent with the single-cell analysis findings, WGCNA revealed a significant positive correlation between monocyte infiltration and both the turquoise module in BC and the blue module in TC. Therefore, these two modules were designated as key consensus gene modules shared between breast and thyroid cancer.

### 3.4 Key consensus module gene signatures differentiate breast and thyroid cancer from healthy controls

**FIGURE 5:**
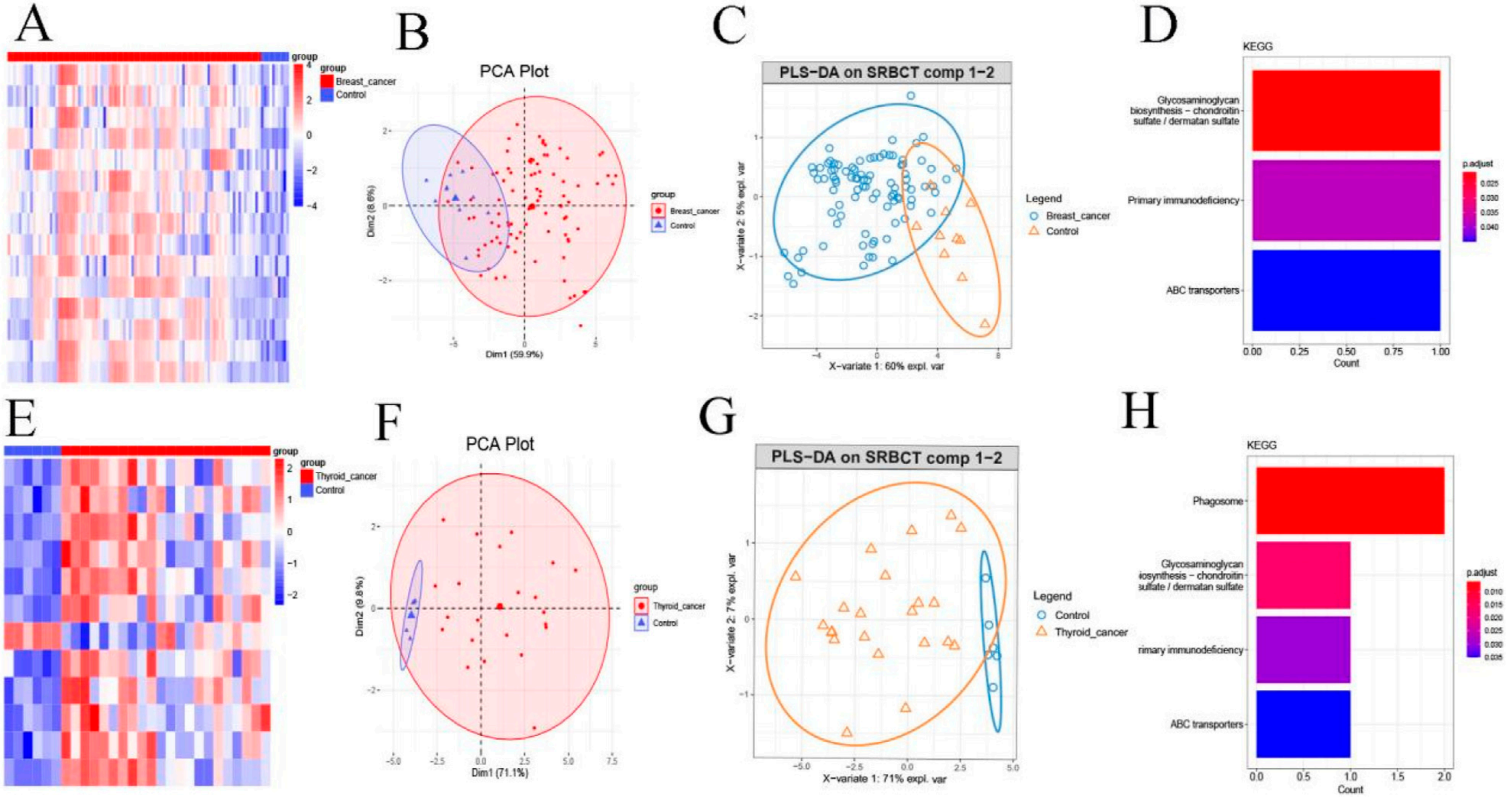
Key Consensus Module Gene Signatures Differentiate Breast and Thyroid Cancer from Healthy Controls. **(A)** Expression heat map of DECs in breast cancer; **(B)** PCA analysis of DEGs in breast cancer; **(C)** PLS-DA analysis of breast cancer; **(D)** KEGG analysis of DEGs in breast cancer; **(E)** Heat map of DEGs in thyroid cancer; **(F)** PCA analysis DEGs in the thyroid cancer; **(G)** PLS-DA analysis of thyroid cancer; **(H)** KEGG analysis of DEGs in the thyroid cancer.

We next evaluated whether the gene signatures from key consensus modules could distinguish patients with BC and TC from healthy individuals. Analyses were performed using Co-DEGs from the blue module of the BC consensus network and the blue module of the TC consensus network. Heatmap visualization revealed two major findings: (1) Co-DEGs were consistently upregulated in both BC and TC patients compared to controls. (2) Hierarchical clustering of key consensus module genes effectively separated cancer patients from healthy controls (Figures 5A,E). Principal PCA based on DEGs from the consensus gene modules of BC and TC demonstrated clear separation between the disease and control groups (Figures 5B,F), with distinct clustering patterns observed for patients with the two cancer types. Additionally, further validation using PLS-DA also revealed different classification patterns between patients with the two cancer types (Figures 5C,G).

To explore potential functional relevance, KEGG pathway enrichment analysis was performed on DEGs from the consensus module of BC and TC. Notably, the glycosaminoglycan biosynthesis pathway was significantly enriched in both cancer types (Figures 5D,H), suggesting a potentially important role for this pathway in the pathogenesis of both BC and TC.

### 3.5 Identification and validation of hub genes shared between breast and thyroid cancer

**FIGURE 6:**
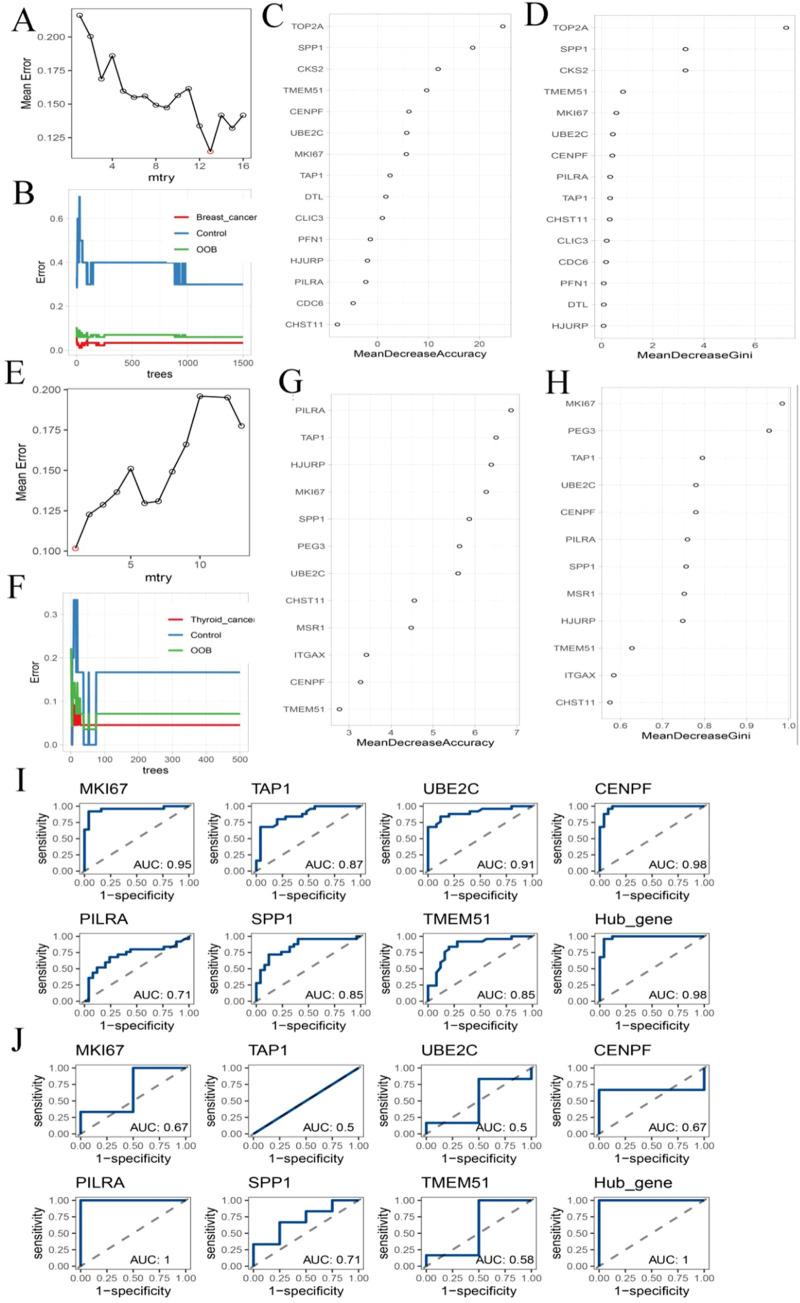
Hub Gene Analysis and Validation. **(A,E)** The change curve of the average error rate of the random forest algorithm as mtry increases; **(B,F)** Error rate fluctuation curve of random forest algorithm with increasing ntrees; **(C,G)** The accuracy ranking of each gene; **(D,H)** The Gini coefficient ranking of each gene; **(I)** The Hub gene’s ranking in breast cancer data ROC analysis; **(J)** ROC analysis of Hub gene in thyroid cancer data.

To identify hub genes within the key consensus modules, DEGs from the BC turquoise module (n = 16) and TC blue module (n = 13) were analyzed using a random forest classifier. The optimal mtry parameter was selected based on the lowest classification error (Figures 6A,E). For BC, the error rate stabilized when the number of decision trees reached 1,500 (Figure 6B), while for TC, stabilization occurred at 500 trees (Figure 6F). These values were used for subsequent analyses, with all other parameters set to default. Based on the Gini coefficient, the top 10 most informative genes for each cancer type were identified (Figures 6C,D,G,H). Intersection of the top-ranked genes from BC (n = 15) and TC (n = 12) yielded seven shared hub genes: MKI67, TAP1, UBE2C, CENPF, PILRA, SPP1, and TMEM51. Receiver operating characteristic (ROC) curve analysis showed that each hub gene exhibited moderate discriminatory ability between cancer patients and healthy controls (Figures 6I,J).

### 3.6 Immune infiltration analysis of hub genes

**FIGURE 7:**
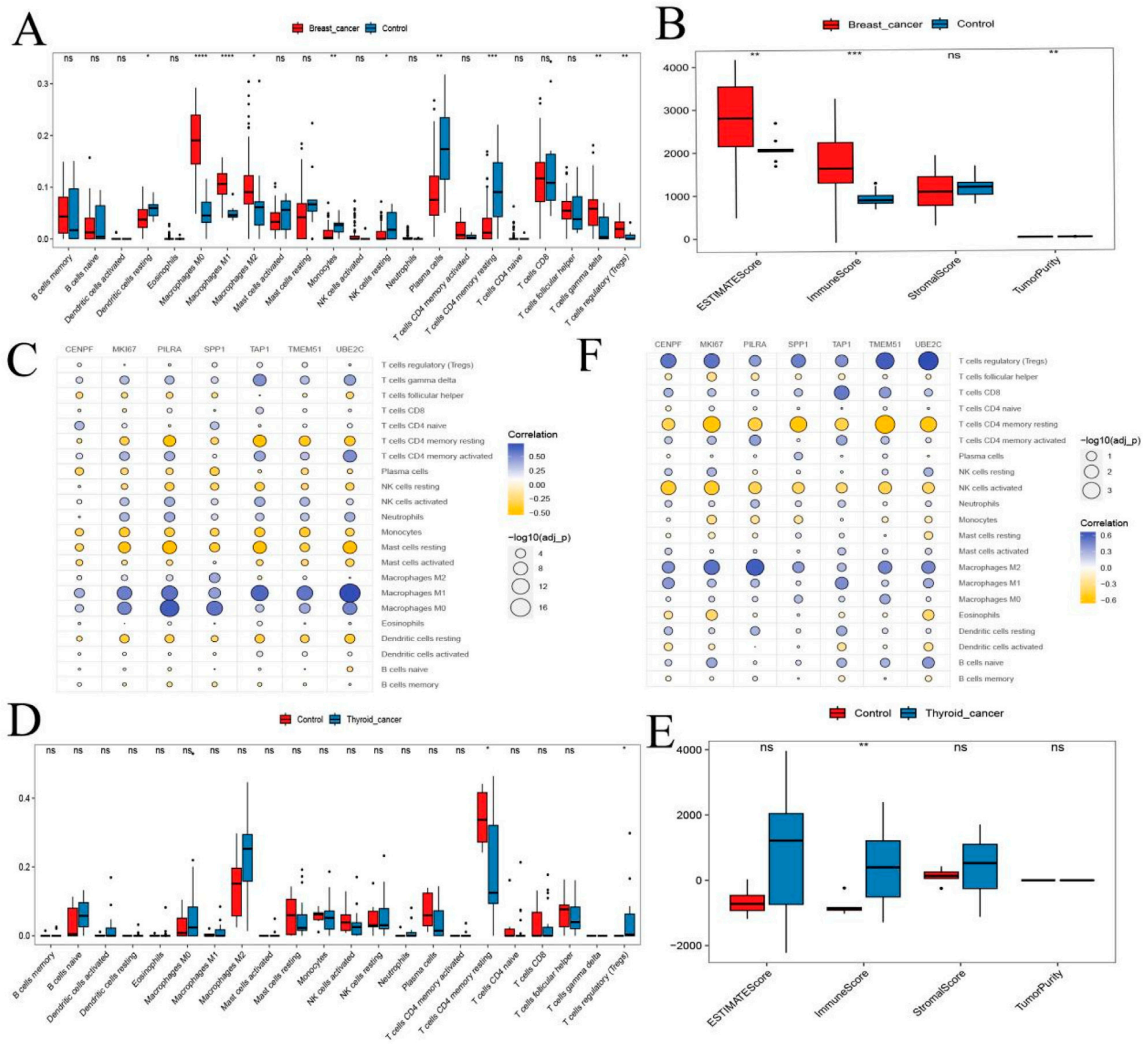
Hub Gene Analysis and Validation. **(A,D)** Box plots of immune infiltration analysis for thyroid cancer and breast cancer; **(B,E)** Analysis of ImmuneScore, StromalScore, ESTIMATEScore, and TumorPurity for thyroid cancer and breast cancer; **(C,F)** Bubble plots showing the correlation between hub genes and immune cells. ns, no statistical difference; *:*p <* 0.05; **:*p <* 0.05; ***:*p <* 0.001.

We further assessed immune infiltration patterns using single-cell transcriptomic data and tumor microenvironment (TME) scoring at the sample level. CIBERSORT analysis revealed significant differences in CD4 memory resting T cells and regulatory T cells (Tregs) between BC and TC samples (Figures 7A,D). ESTIMATE analysis also indicated significant variation in ImmuneScore between BC and TC (Figures 7B,E). These findings suggest that T cell populations may play a central role in shaping the immune landscape in both cancers.

Spearman correlation analysis between the seven hub genes and immune cell proportions showed that, in both BC and TC: Hub genes were negatively correlated with CD4 memory resting T cells and monocytes. Hub genes were positively correlated with Tregs and M1 macrophages (Figures 7C,F). These results suggest that hub genes may influence BC and TC progression through modulation of immune cell infiltration.

### 3.7 Transcription factor and miRNA regulatory network analysis of hub genes

**FIGURE 8:**
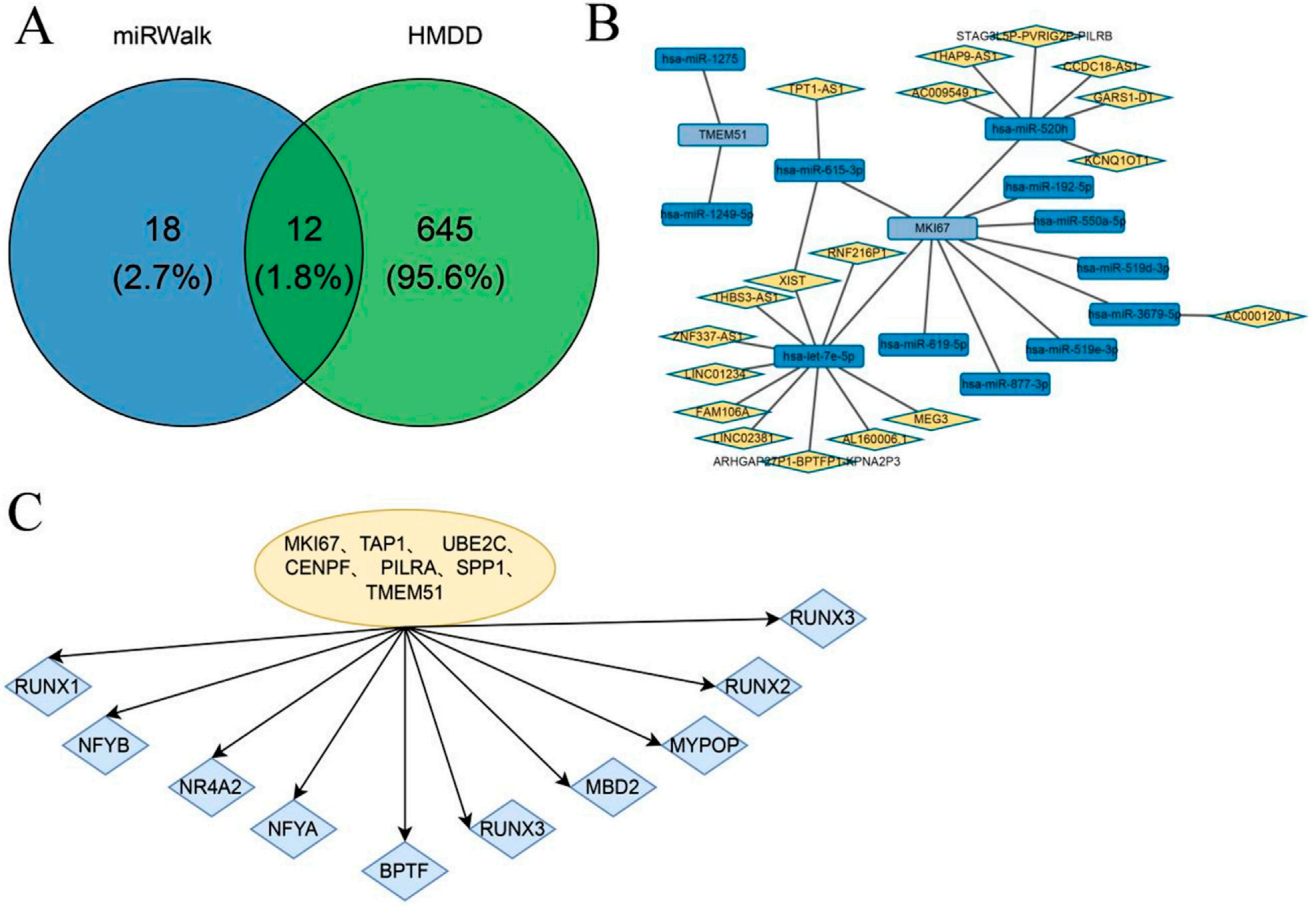
TF and miRNA Regulatory Network Analysis of Hub Genes. **(A)** Breast cancer and thyroid cancer-related miRNAs and the Venn diagram of miRNAs related to Hub genes extracted from the miRWalk database; **(B)** The miRNA network of Hub genes; **(C)** Transcription factor enrichment analysis of Hub gene.

To investigate potential regulatory mechanisms underlying the expression of the identified hub genes, we analyzed both TF and miRNA regulatory networks. Tumor-associated miRNAs were obtained from the Human miRNA Disease Database (HMDD). mRNA–miRNA interaction pairs involving hub genes were extracted from the miRWalk database and intersected with 657 tumor-related miRNAs, resulting in 12 validated mRNA–miRNA interaction pairs (Figure 8A). Using the ENCORI database, we then predicted lncRNAs interacting with these tumor-associated miRNAs, and corresponding interaction pairs were identified (Figure 8B). In parallel, motif enrichment analysis was performed to identify key TFs potentially regulating the hub genes. The results suggested that hub gene expression may be regulated by transcription factors such as NFYB, NR4A2, and BPTF (Figure 8C).

### 3.8 Validation of hub gene expression

**FIGURE 9:**
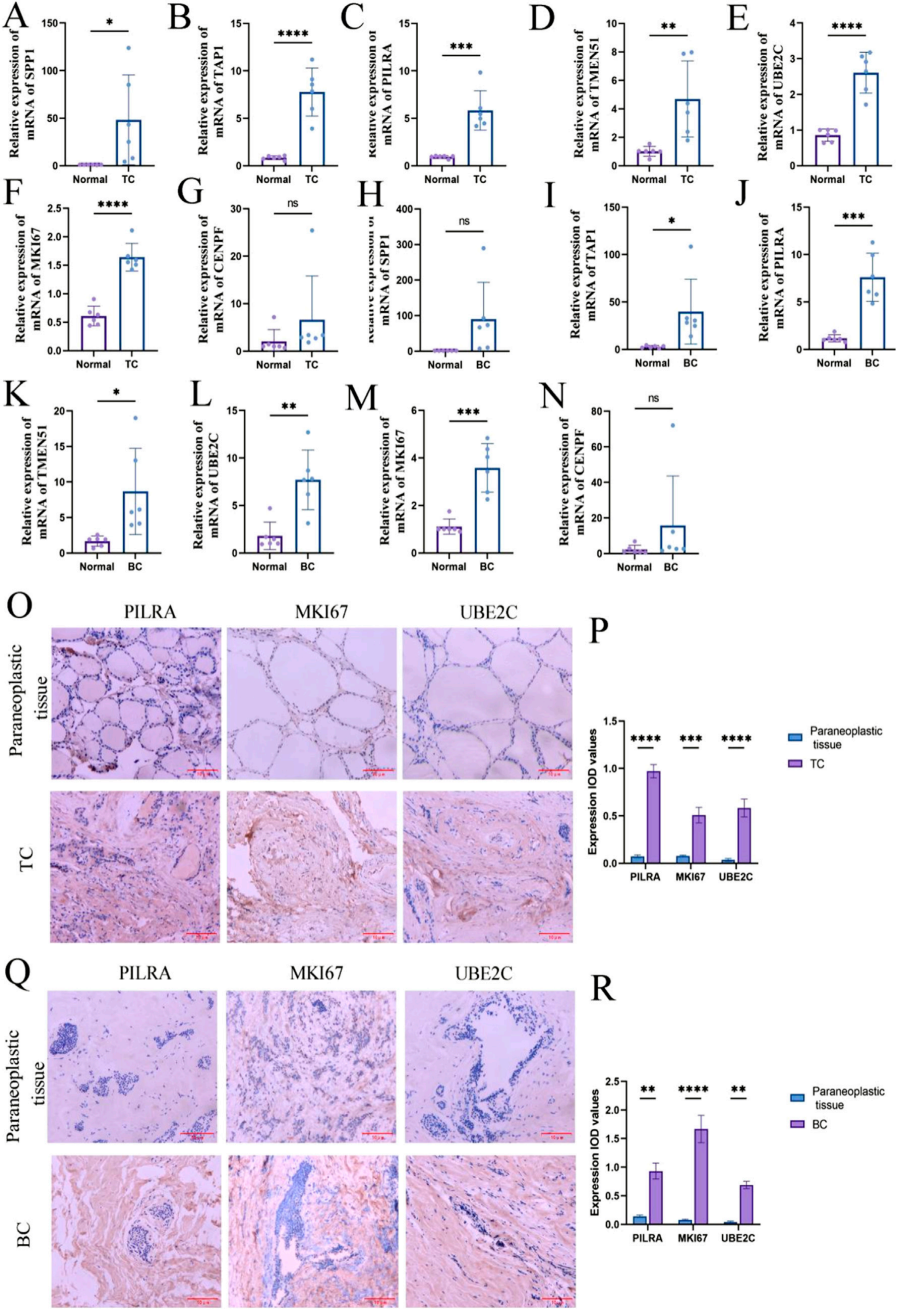
Hub gene expression level. **(A–G)** RT-qPCR to detect the expression level of Hub gene in thyroid cancer; **(H–P)** RT-qPCR to detect the expression level of Hub gene in breast cancer. **(Q)** Immunohistochemistry to detect the expression level of PILRA, MKI67, UBE2C in thyroid cancer and paracancerous tissues. **(R)** Immunohistochemistry to detect the expression level of PILRA, MKI67, UBE2C in breast cancer and paracancerous tissues. ns, no statistical difference; *: *p <* 0.05; **: *p <* 0.05; ***: *p <* 0.001; ****: *p <* 0.0001.

To validate hub gene expression, we performed RT-qPCR on tumor and adjacent normal tissue samples. In TC tissues, the expression levels of SPP1, TAP1, PILRA, TMEM51, UBE2C, and MKI67 were significantly upregulated compared to adjacent normal tissues, whereas CENPF did not show a statistically significant difference (Figures 9A–G). In BC tissues, TAP1, PILRA, UBE2C, TMEM51, and MKI67 were significantly upregulated, while the expression levels of SPP1 and CENPF remained unchanged between cancer and adjacent tissues (Figures 9H–N). Subsequently, we selected PILRA, MKI67, and UBE2C, which were significantly upregulated in both cancer types, for further validation using immunohistochemistry. The IHC results confirmed that the protein levels of these three genes were significantly elevated in both BC and TC tissues compared to adjacent normal tissues (Figures 8O–R).

## 4 Discussion

BC and TC are the two most prevalent malignancies among women, and increasing evidence indicates a bidirectional association between them. Epidemiological studies have shown that women diagnosed with TC have a higher risk of subsequently developing BC, and *vice versa*, suggesting the existence of shared etiological factors (Bolf et al., 2019). Although this co-occurrence has been reported worldwide, the underlying molecular mechanisms remain largely unexplored. Therefore, investigating the common pathogenic pathways and potential therapeutic targets shared by BC and TC is of vital importance.

In this study, we integrated scRNA-seq and bulk transcriptomic data to explore shared molecular mechanisms and therapeutic targets in BC and TC. Transcriptome analysis identified 76 shared differentially expressed genes (Co-DEGs). Combined scRNA-seq and deconvolution analyses further revealed that monocyte infiltration is significantly enriched in both cancers, highlighting a shared immune microenvironment component. Using WGCNA and integrating immune infiltration features, we identified two consensus gene modules—the turquoise module in BC and the blue module in TC—as key regulatory units. A subsequent random forest classifier identified seven hub genes shared by BC and TC: MKI67, TAP1, UBE2C, CENPF, PILRA, SPP1, and TMEM51. Among these, PILRA, MKI67, and UBE2C showed consistently elevated expression in both cancers and were validated through RT-qPCR and immunohistochemistry, suggesting their potential as therapeutic targets.

PILRA (Paired Immunoglobulin-like Type 2 Receptor Alpha) is an immune-inhibitory receptor containing two immunoreceptor tyrosine-based inhibitory motifs (ITIMs) and is mainly expressed in monocytes, dendritic cells, and granulocytes (Lu et al., 2014; Kogure et al., 2011). Prior research has shown its involvement in regulating immune cell infiltration and promoting inflammatory responses (Shi et al., 2023). In this study, elevated PILRA expression in both BC and TC coincided with increased monocyte infiltration, supporting the hypothesis that PILRA may mediate tumor progression through immune regulation.

Mki67, also known as Ki67, is an excellent marker of active cell proliferation in normal and tumor cell populations (Schlüter et al., 1993). Very low levels of Ki67 have been reported in normal healthy breast tissue (Loibl et al., 2021). And the expression of Ki67 is significantly higher in proliferatively enlarged lobular units than in adjacent normal terminal ductal lobular units (Lee et al., 2006) and is associated with subsequent breast cancer risk (Zhang et al., 2021). However, Ki67 has limited use in thyroid cancer pathology compared to breast cancer (Agarwal et al., 2021). Nevertheless, he can also distinguish between non-neoplastic and neoplastic thyroid lesions.

UBE2C, an E2 ubiquitin-conjugating enzyme, is widely recognized for its role in tumor progression and poor prognosis across various cancers (Huang et al., 2021; Presta et al., 2020). In BC, UBE2C overexpression is linked to higher histological grade, lymphovascular invasion, and early metastasis. Mechanistically, UBE2C knockdown restores PTEN expression and suppresses the AKT/mTOR/HIF-1α pathway, thereby reducing proliferation and invasiveness (Guo et al., 2023; Zheng et al., 2023). Although less studied in TC, recent findings suggest that UBE2C knockdown can suppress TC cell proliferation and migration while enhancing chemosensitivity (Xiang and Yan, 2022).

In addition, KEGG pathway enrichment analysis of the consensus gene modules revealed significant enrichment in the glycosaminoglycan (GAG) biosynthesis pathway. GAGs are long-chain, highly sulfated polysaccharides (e.g., heparan sulfate, chondroitin sulfate, dermatan sulfate, hyaluronic acid) synthesized by specific glycosyltransferases (Wieboldt and Läubli, 2022). They regulate growth factor signaling, ECM remodeling, and tumor metastasis. Abnormal GAG accumulation, especially of chondroitin sulfate, is associated with poor prognosis in both BC and TC (Yen et al., 2024; Sui et al., 2024), while SDC-1, a heparan sulfate proteoglycan, promotes invasion via cell-cell and cell-ECM adhesion (Bologna-Molina et al., 2010; Hassan et al., 2023). These findings highlight the glycosaminoglycan pathway as a promising therapeutic targe.

In summary, we identified three core genes (PILRA, MKI67, UBE2C) as potential therapeutic targets in BC and TC. *In vitro* validation supported their elevated expression and clinical relevance. However, our study has certain limitations. Although key genes were identified, the exact molecular mechanisms through which they influence tumor progression remain to be elucidated. Furthermore, due to dataset constraints, we were unable to conduct subgroup analyses (e.g., based on hormone receptor status or cancer subtype), which may affect the generalizability of the results. In future studies, we plan to collect clinical samples from patients with secondary co-occurrence of BC and TC, enabling a more precise evaluation of core gene pathways in disease development and prognosis. This will enhance the clinical translation of our findings and potentially inform targeted treatment strategies.

## 5 Conclusion

In summary, this study suggests that PILRA, MKI67, and UBE2C may serve as both diagnostic biomarkers and therapeutic targets for breast and thyroid cancers. These findings not only enhance our understanding of the shared molecular mechanisms underlying the co-morbidity of these two malignancies but also offer valuable insights for the development of targeted clinical therapies.

## Data Availability

The dataset utilized in this study is available in an online repository. The repository name and access number are provided within the article. Additionally, the R scripts used for the analysis are available in the Supplementary Material.
