# Supplementary material for: Single-cell RNA-seq combined with bulk RNA-seq explores shared gene signatures between thyroid and breast cancers
Source: Front Genet. 2025 Nov 17;16:1609189. doi: 10.3389/fgene.2025.1609189 (PMC12665382; doi:10.3389/fgene.2025.1609189)
Supplement: Supplementary file 1 [file DataSheet1.docx]

All parameters used in the analysis process of this study are default parameters. The R packages and environment information used are as follows：

> sessionInfo()

R version 4.1.2 (2021-11-01)

Platform: x86_64-pc-linux-gnu (64-bit)

Running under: Debian GNU/Linux 11 (bullseye)

Matrix products: default

BLAS: /Pub/Apps/Cellar/R/4.1.2/lib/R/lib/libRblas.so

LAPACK: /Pub/Apps/Cellar/R/4.1.2/lib/R/lib/libRlapack.so

locale:

[1] LC_CTYPE=en_US.UTF-8 [2]LC_NUMERIC=C

[3] LC_TIME=en_US.UTF-8 [4]LC_COLLATE=en_US.UTF-8

[5] LC_MONETARY=en_US.UTF-8 [6] LC_MESSAGES=en_US.UTF-8

[7] LC_PAPER=en_US.UTF-8 [8]LC_NAME=C

[9] LC_ADDRESS=C [10]LC_TELEPHONE=C

[11] LC_MEASUREMENT=en_US.UTF-8 LC_IDENTIFICATION=C

attached base packages:

[1] stats graphics grDevices utils datasets methods base

other attached packages:

[1] RcisTarget.hg19.motifDBs.cisbpOnly.500bp_1.14.0

[2] RcisTarget_1.14.0

[3] DT_0.29

[4] pROC_1.18.4

[5] ggpubr_0.4.0

[6] randomForest_4.7-1.1

[7] cowplot_1.1.3

[8] FactoMineR_2.8

[9] factoextra_1.0.7

[10] WGCNA_1.72-1

[11] fastcluster_1.2.3

[12] dynamicTreeCut_1.63-1

[13] lubridate_1.9.2

[14] forcats_1.0.0

[15] stringr_1.5.0

[16] dplyr_1.1.4

[17] purrr_1.0.2

[18] readr_2.1.4

[19] tidyr_1.3.0

[20] tibble_3.2.1

[21] ggplot2_3.5.0

[22] tidyverse_2.0.0

loaded via a namespace (and not attached):

[1] backports_1.4.1 Hmisc_5.1-1

[3] plyr_1.8.8 GSEABase_1.56.0

[5] splines_4.1.2 GenomeInfoDb_1.30.1

[7] TH.data_1.1-2 digest_0.6.33

[9] foreach_1.5.2 htmltools_0.5.7

[11] GO.db_3.14.0 fansi_1.0.6

[13] magrittr_2.0.3 checkmate_2.2.0

[15] memoise_2.0.1 cluster_2.1.4

[17] doParallel_1.0.17 tzdb_0.4.0

[19] Biostrings_2.62.0 annotate_1.72.0

[21] matrixStats_1.0.0 R.utils_2.12.2

[23] sandwich_3.0-2 timechange_0.2.0

[25] colorspace_2.1-0 blob_1.2.4

[27] ggrepel_0.9.3 xfun_0.43

[29] crayon_1.5.2 RCurl_1.98-1.6

[31] jsonlite_1.8.7 graph_1.72.0

[33] impute_1.68.0 survival_3.5-7

[35] zoo_1.8-12 iterators_1.0.14

[37] glue_1.6.2 gtable_0.3.4

[39] zlibbioc_1.40.0 emmeans_1.8.8

[41] XVector_0.34.0 DelayedArray_0.20.0

[43] car_3.1-2 BiocGenerics_0.40.0

[45] abind_1.4-5 scales_1.3.0

[47] mvtnorm_1.2-3 DBI_1.1.3

[49] rstatix_0.7.2 Rcpp_1.0.12

[51] xtable_1.8-4 htmlTable_2.4.1

[53] flashClust_1.01-2 foreign_0.8-85

[55] bit_4.0.5 preprocessCore_1.56.0

[57] Formula_1.2-5 stats4_4.1.2

[59] htmlwidgets_1.6.2 httr_1.4.7

[61] ellipsis_0.3.2 pkgconfig_2.0.3

[63] XML_3.99-0.14 R.methodsS3_1.8.2

[65] nnet_7.3-19 multcompView_0.1-9

[67] utf8_1.2.4 tidyselect_1.2.0

[69] rlang_1.1.4 later_1.3.1

[71] AnnotationDbi_1.56.2 munsell_0.5.0

[73] tools_4.1.2 cachem_1.0.8

[75] cli_3.6.1 generics_0.1.3

[77] RSQLite_2.2.9 broom_1.0.5

[79] evaluate_0.21 fastmap_1.1.1

[81] knitr_1.40 bit64_4.0.5

[83] KEGGREST_1.34.0 mime_0.12

[85] R.oo_1.25.0 arrow_13.0.0

[87] leaps_3.1 compiler_4.1.2

[89] rstudioapi_0.15.0 png_0.1-8

[91] ggsignif_0.6.4 stringi_1.7.8

[93] lattice_0.21-8 Matrix_1.5-3

[95] vctrs_0.6.4 pillar_1.9.0

[97] lifecycle_1.0.4 estimability_1.4.1

[99] data.table_1.14.8 bitops_1.0-7

[101] httpuv_1.6.5 AUCell_1.16.0

[103] GenomicRanges_1.46.1 R6_2.5.1

[105] promises_1.2.1 gridExtra_2.3

[107] IRanges_2.28.0 codetools_0.2-19

[109] assertthat_0.2.1 MASS_7.3-60.0.1

[111] SummarizedExperiment_1.24.0 withr_3.0.0

[113] multcomp_1.4-25 S4Vectors_0.32.4

[115] GenomeInfoDbData_1.2.7 parallel_4.1.2

[117] hms_1.1.3 grid_4.1.2

[119] rpart_4.1.16 coda_0.19-4

[121] rmarkdown_2.24 MatrixGenerics_1.6.0

[123] carData_3.0-5 scatterplot3d_0.3-44

[125] Biobase_2.54.0 shiny_1.7.5

[127] base64enc_0.1-3
